# Exercise tests in Chagas cardiomyopathy: an overview of functional evaluation, prognostic significance, and current challenges

**DOI:** 10.1590/0037-8682-0100-2020

**Published:** 2020-07-03

**Authors:** Henrique Silveira Costa, Márcia Maria Oliveira Lima, Pedro Henrique Scheidt Figueiredo, Vanessa Pereira Lima, Matheus Ribeiro Ávila, Kenia Kiefer Parreiras de Menezes, Vanessa Amaral Mendonça, Ana Cristina Rodrigues Lacerda, Maria Carmo Pereira Nunes, Mauro Felippe Felix Mediano, Manoel Otávio da Costa Rocha

**Affiliations:** 1 Universidade Federal dos Vales do Jequitinhonha e Mucuri, Faculdade de Ciências Biológicas e da Saúde, Departamento de Fisioterapia, Diamantina, MG, Brasil.; 2 Universidade Federal de Minas Gerais, Escola de Medicina, Curso de pós-graduação em Infectologia e Medicina Tropical, Belo Horizonte, MG, Brasil.; 3 Universidade Federal de Minas Gerais, Escola de Educação Física, Fisioterapia e Terapia Ocupacional, Curso de pós-graduação em Ciências da Reabilitação, Belo Horizonte, MG, Brasil.; 4 Fundação Oswaldo Cruz, Instituto Nacional de Infectologia Evandro Chagas, Rio de Janeiro, RJ, Brasil.

**Keywords:** Chagas disease, Chagas cardiomyopathy, Exercise test, Exercise tolerance, Evaluation, Prognosis

## Abstract

Patients with Chagas cardiomyopathy (ChC) usually progress with fatigue and dyspnea. Exercise tests are valuable for the functional evaluation of these patients. However, information about the applicability of the exercise tests is scattered, and no studies have systematically reviewed the results. Thus, the present review explored the general aspects and prognostic value of exercise tests in patients with ChC. A literature search of the MEDLINE, Web of Science, CINAHL, Scopus, and LILACS databases was performed to identify relevant studies. There were no data restrictions, and articles that met the objective of the study were selected. Articles written in English, Portuguese, and Spanish were considered, and 25 articles were finally included. The peak oxygen uptake (VO2peak) was correlated with demographic and echocardiographic variables. Echocardiographic features of the left ventricular diastolic function and right ventricular systolic function appeared to be determinants of functional capacity, in addition to age and sex. VO2peak was associated with higher mortality, especially in patients with dilated ChC. The minute ventilation/carbon dioxide production slope (VE/VCO2 slope) was a strong predictor of survival; however, more studies are needed to verify this observation. Field tests showed moderate to strong correlation with VO2peak and thus may be inexpensive tools for the functional evaluation of patients with ChC. However, few studies have verified their prognostic significance. While exercise tests are useful tools for functional assessment, information is scarce regarding further considerations, and many of the criteria are based on guidelines for other heart diseases.

## INTRODUCTION

Chagas disease remains a serious public health problem. While the incidence and prevalence are decreasing dramatically, 6 million people are infected in Latin America and more than 70 million are at a risk of infection^1^. In Brazil, Chagas disease is a major cause of morbidity and mortality among tropical diseases and accounts for approximately 7.5 times as many disability-adjusted life-years lost as malaria^2^. Among infected patients, 30-40% develop a cardiac form of the disease^3^ ^,^ ^4^, with a wide spectrum of manifestations such as clinically nonapparent abnormalities, severe heart failure, thromboembolism, malignant arrhythmias, and sudden cardiac death^5^. A recent statement^6^ standardized the terms Chagas cardiomyopathy (ChC) to define patients with cardiac involvement (electrocardiographic abnormality in patients with positive serological tests against *Trypanosoma cruzi*) and dilated ChC to describe patients with left ventricular enlargement with systolic dysfunction. However, patients with ChC, regardless of systolic function and degree of ventricular dilation, have impaired functional capacity^7^, which reinforces the need for evaluation by exercise tests.

Even in asymptomatic patients, non-invasive methods such as conventional maximal exercise test and cardiopulmonary exercise testing (CPET) can detect significant changes, including exercise-induced ventricular arrhythmias (EIVAs)^8^ and chronotropic incompetence^9^. However, the usefulness of exercise tests in ChC has not been systematically discussed. This review explored the applicability of exercise tests in patients with ChC, highlighting their general aspects, determinants, and prognostic value, as well as the challenges faced.

## SEARCH METHOD

This comprehensive review aimed to verify the applicability and prognostic value of exercise tests for the functional evaluation of patients with ChC. Relevant studies were identified from searches of the Medical Literature Analysis and Retrieval System Online (MEDLINE), Web of Science, the Cumulative Index to Nursing and Allied Health Literature (CINAHL), Scopus, and Latin American & Caribbean Health Sciences Literature (LILACS) databases using search terms related to ChC, functional capacity, and exercise tests.

The eligibility criteria included studies that a) enrolled patients diagnosed with ChC; b) evaluated functional capacity using CPET, conventional exercise tests, or field tests; and c) were written in the English, Spanish, or Portuguese. We defined ChC with preserved left ventricular ejection fraction (LVEF) as those patients with positive serology for *Trypanosoma cruzi*, a normal ventricular function, and the presence of arrhythmias and/or conduction disorders. Patients with left ventricular systolic dysfunction and cardiac dilation were categorized as having dilated ChC^10^. The exclusion criteria were review studies, duplicate articles, animal studies, manuscripts that compared functional parameters to other heart conditions, articles that did not demonstrate the clinical presentation of ChC, and articles that did not match the objective of the present review. Manuscripts without statistical analysis, as well as those with fewer than 10 individuals per group, were also excluded. There were no restrictions on the year of publication until January 2020. This review did not include comparisons of functional capacity between healthy individuals and patients with ChC as a recent systematic review and meta-analysis^7^ has already been performed.

**FIGURE 1:**
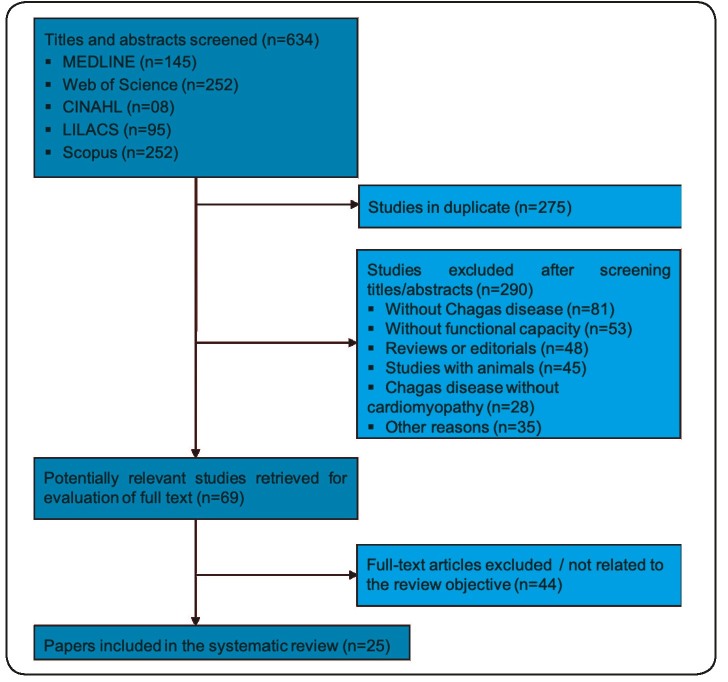
Flow of studies through the review. **MEDLINE:** Medical Literature Analysis and Retrieval System Online; **CINAHL:** Cumulative Index to Nursing and Allied Health Literature; **LILACS:** Latin American & Caribbean Health Sciences Literature.

The original search identified 634 articles. After reading the titles and abstracts of these articles to identify all terms related to functional evaluation by exercise tests, 69 were selected for full-text review and 25 articles met the criteria for this investigation. Figure 1 outlines the flow of papers through the review.

## GENERAL ASPECTS OF FUNCTIONAL EVALUATION BY MAXIMAL EXERCISE TESTS

Maximal exercise tests are widely used in the functional assessment of patients with ChC. The CPET is increasingly performed for patients with heart disease worldwide^11^ and is considered the gold standard for functional evaluation with significant prognostic value^11^ ^-^ ^13^. The major variables with functional and clinical significance obtained during the CPETs are peak oxygen uptake (VO2peak) and the slope of the increase of ventilation relative to carbon dioxide production (VE/VCO2 slope)^13^.

The VO2peak, defined as the highest VO_2_ value reached during the exercise test, is used as a primary outcome in evaluating exercise capacity and the effectiveness of rehabilitation programs^14^ ^,^ ^15^. In addition to VO2peak, the VE/VCO2 slope is used to analyze ventilatory response during exercise and has been recommended as a parameter for the functional evaluation of patients with heart failure^16^. The VE/VCO2 slope represents the efficiency of carbon dioxide elimination during physical exertion^17^. Another parameter, the anaerobic threshold (AT), reflects the functional capacity at submaximal intensity (many cardiac patients cannot reach maximum intensity) and does not depend on patient motivation^18^. 

Unlike the CPET, in which VO2peak is directly measured by gas analysis, the conventional exercise test uses a formula to estimate the VO2peak. Despite the potential discrepancies between estimated and direct measurements of VO2peak, both methods aid in the prediction of cardiovascular risk and mortality^19^. Additionally, EIVA is a marker of cardiovascular mortality in patients with ChC^20^. As EIVA occurs frequently in ChC patients without apparent cardiac involvement^8^, the conventional maximal exercise test is clinically relevant for risk stratification in this population.

Nine articles on ChC reported the general aspects of functional evaluation by maximum tests and their determinants (Table 1). One study^21^ demonstrated no difference in VO2peak between groups with dilated ChC and with preserved LVEF. However, the study evaluated VO2peak using the conventional maximum exercise test, which could misreport the result. To clarify the hypothesis, two other studies compared functional capacities between the two clinical presentations of ChC using the CPET. Both found significant functional differences between groups. Mady et al.^22^ reported significantly lower VO2peak, O2 pulse, heart rate, minute ventilation (VE), and volume of exhaled carbon dioxide (VCO2) in patients with dilated ChC than in patients with preserved LVEF. Similarly, using CPET, Costa et al.^23^ demonstrated that lower VO2peak values and higher VE/VCO2 slope values were observed in patients with dilated ChC than in those with preserved LVEF. Previous findings suggest that the use of more accurate assessment methods will allow the detection of important functional changes in patients with dilated ChC compared to those in patients with preserved LVEF.

**TABLE 1:** General aspects of the included studies (n=9) that used the maximum exercise test for the functional evaluation of patients with ChC.

STUDY	POPULATION	EXERCISE TEST	RESULTS
Mady et al. (1986)^22^	13 patients with ChC and preserved LVEF (30±5.76 years) and 15 patients with dilated ChC (30±6.82 years, NYHA II-III)	CPET (Naughton protocol, treadmill)	The VO2peak, O2 pulse, HR, VE, and VCO2 in patients with dilated ChC were significantly lower compared to those in patients with ChC and preserved LVEF (p<0.05).
Pedrosa; Campos (2004)^33^	20 patients with ChC and preserved LVEF (30% male, 51.2±11 years, LVEF: 59.3±14%) and 20 patients with dilated ChC (20% male, 55.8±10 years, LVEF: 37.6±9.3%)	CPET (Bruce protocol, treadmill)	No differences (p>0.05) were observed in the prevalence of ventricular extrasystoles, pairs of extrasystoles, nonsustained ventricular tachycardia, and sustained monomorphic ventricular tachycardia, detected by 24-h Holter monitoring and those induced by exercise both in dilated ChC and with preserved LVEF.
Rocha et al. (2005)^21^	154 patients with ChC and preserved LVEF [57% male, 41.7±9.3 years, LVEF: 62 (58-65)%] and 17 patients with dilated ChC [59% male, 42.8±9.2 years, LVEF: 35 (31-39)%]	Maximal Exercise Testing (Bruce protocol, treadmill)	No differences were observed between groups with preserved LVEF and dilated ChC in VO2peak, maximum HR, delta HR, and effort time (p>0.05).
Lima et al. (2010)^25^	40 patients with dilated ChC; 49±8 years; 58% male; NYHA I-II; LVEF: 36.3±7.8%	Maximal Exercise Testing (Bruce protocol, treadmill)	The VO2peak was correlated with E/E’ ratio (r= -0.516; p=0.001) but not with LVEF, LVDd, and BNP. The predictors of VO2peak in the final multivariate model were age, female sex, E/E’ ratio, and left atrial volume (LAV) (r^2^=0.521).
Nunes et al. (2010)^27^	65 patients with ChC (comprising dilated ChC and with preserved LVEF); 48.6±9.1 years; 60% male; NYHA I-II); LVEF: 43.1±11.4%	Maximal Exercise Test (treadmill, Bruce protocol)	The VO2peak was correlated with LVDd/BSA (r= -0.38; p=0.002), e’ (r= 0.40; p=0.001), E/e’ ratio (r= -0.37; p=0.003), RV e’ (r= 0.29; p=0.025), RV e’/A’ ratio (r= 0.41; p=0.001), RV systolic velocity (r= 0.45; p<0.001), RV Tei index (r= -0.28; p=0.029), and PASP (r= -0.36; p=0.009). The VO2peak did not correlate with LVEF, E/A ratio, DT, LA volume index, E/Vp. The independent predictors of VO2peak were age, female sex and RV systolic velocity (r^2^=0.71).
Alvarenga et al. (2014)^24^	35 patients with ChC (dilated and with preserved LVEF); 47.11±8.15 years, 65% male; NYHA I-III; LVEF: 59.0 [41.0-64.0]	CPET (ramp protocol, treadmill)	In the overall study population, a significant correlation was observed between VO2peak and LVEF (r=0.536, p=0.001) and E/e' ratio (r=-0.399; p=0.022). In patients with dilated ChC (n=16), the VO2peak was also correlated with LVEF (r=0.611, p=0.016) and with the ratio E/e' (r=-0.601, p=0.018). In the multivariate analysis, LVEF and E/e’ ratio were strong predictors of VO2peak (r^2^ = 0.723) only in patients with dilated ChC.
Costa et al. (2014)^23^	41 patients with ChC (dilated and with preserved LVEF); 47.8±8.3 years; 68% male; NYHA I-III	CPET (ramp protocol, treadmill)	Patients with dilated ChC had lower VO2peak (p=0.001) and 6MWT distance (p=0.045) values and higher VE/VCO2 slope (p=0.029) value compared to those in patients with ChC and preserved LVEF.
Costa et al. (2017)^26^	81 patients with ChC (dilated and with preserved LVEF); 48.6±8.1 years; 63% male; NYHA I-III); LVEF: 43.7±13.7%	Maximal Exercise Test (treadmill)	The VO2peak was correlated with age (r= -0.490; p<0.001), sex (r = 0.283; p=0.010), body mass index (r= -0.333; p=0.002), NYHA functional class (r= -0.667; p<0.001), and 6MWT distance (r=0.527; p<0.001). The VO2peak was predicted by sex, NYHA functional class, 6MWT distance, age, and body mass index
Costa et al. (2017)^28^	48 patients with ChC (dilated and with preserved LVEF); 56.4 (53.3-59.5) years; 29% male; NYHA I-III; LVEF: 54.3 (48.6-59.9)%	CPET (ramp protocol, treadmill)	VE/VCO2 slope was correlated with the percentage of maximal inspiratory pressure (r= -0.446; p=0.004). The VO2peak was not correlated with maximal inspiratory pressure. In the final multivariate model, patients with impaired VE/VCO2 slope had a 1.2-fold increased risk for inspiratory muscle weakness (prevalence ratio 1.2, 95% CI 1.1 to 1.5, p = 0.001).

Data presented as mean±standard deviation; median (25-75%); mean [95% CI] or percentage. **ChC:** Chagas cardiomyopathy; **NYHA:** New York Heart Association; **CPET:** cardiopulmonary exercise testing; **LVEF:** left ventricular ejection fraction; **VO2peak:** peak oxygen uptake; **HR:** heart rate; **VE:** minute-ventilation; **LVDD:** left ventricular end-diastolic diameter; **BSA:** body surface area; **E:** early diastolic transmitral flow velocity; **e´:** early diastolic mitral annular velocity; **E/A**: ratio of early to late transmitral flow velocity; **E/e´ ratio**: ratio of the early diastolic transmitral flow velocity to early diastolic mitral annular velocity; **E/Vp:** ratio of the early diastolic transmitral flow velocity to color M-mode flow propagation velocity; **PASP:** pulmonary artery systolic pressure; **RV:** right ventricular; **DT:** deceleration time; **LA:** left atrium; **VE/VCO2 slope:** minute ventilation/carbon dioxide production slope; **6MWT:** Six-minute walk test.

Correlation analyses between VO2peak and other variables of clinical importance in ChC were also identified. Only one^24^ of the included studies used CEPT to evaluate VO2peak, which was negatively correlated with the ratio of the early diastolic transmitral flow velocity to early diastolic mitral annular velocity (E/e’ ratio) and was positively correlated with LVEF. When the sample was stratified according to dilated ChC and with preserved LVEF, the correlation between the VO2peak and LVEF and E/e’ ratio remained only in the dilated group.

The VO2peak in the conventional maximal exercise test was negatively correlated with age^25^ ^,^ ^26^, body mass index^26^, female sex^26^, the New York Heart Association (NYHA) class^26^, and echocardiographic parameters (left ventricular end-diastolic diameter indexed by body surface area, deceleration time, E/e’ ratio, right ventricle Tei index, and pulmonary artery systolic pressure)^25^ ^,^ ^27^. Furthermore, the VO2peak was positively correlated with echocardiographic features, including e’^25^ ^,^ ^27^, right ventricle e’^27^, right ventricle e’/A’ ratio^27^, and right ventricle systolic velocity^27^.

Despite its clinical importance, the general aspects of the VE/VCO2 slope in patients with ChC remain undetermined. One study^28^ demonstrated a correlation between VE/VCO2 slope and inspiratory muscle strength and observed a 1.2-fold increased risk of inspiratory muscle weakness in patients with impaired VE/VCO2. As it is an important variable in the functional and prognostic evaluation of patients with heart failure^29^ ^-^ ^32^, further studies are needed to verify the value of VE/VCO2 slope in patients with ChC.

EIVA is one of the most important findings of the maximal exercise test as it can reflect electrocardiographic patterns during exertion. Pedrosa and Campos^33^ observed no differences in the prevalence of ventricular extrasystoles, pairs of extrasystoles, and non-sustained ventricular tachycardia and sustained monomorphic ventricular tachycardia detected by 24-h Holter monitoring and those induced by exercise in ChC. Since the maximal exercise test showed results similar to those of 24-h Holter monitoring, which detects spontaneous arrhythmias, the test can be considered safe for patients.

## DETERMINANTS OF FUNCTIONAL CAPACITY

Studies have verified the determinants of functional capacity in patients with ChC. One such study^26^ in patients with dilated ChC and preserved LVEF reported that male sex, NYHA class, six-minute walk test (6MWT) distance, age, and body mass index were independent determinants of VO2peak, while echocardiographic findings were not.

Three studies^24^ ^,^ ^25^ ^,^ ^27^ assessed whether parameters evaluated on echocardiography were independent determinants of VO2peak. One of these studies^24^ reported the determinants of VO2peak evaluated by gas analysis in patients with ChC after stratifying the sample according to left systolic function and ventricular dilatation. They found that LVEF and E/e’ ratio combined were strong determinants of VO2peak only in patients with dilated ChC. However, the sample size was small, and thus, the results should be interpreted with caution. Another study^25^ of the determinants of VO2peak in patients with dilated ChC found that age, male sex, E/e’ ratio, and left atrial volume were independently associated with functional capacity. As in the aforementioned study^24^, the E/e’ ratio remained a determinant of functional capacity in patients with dilated ChC. However, LVEF was not associated with functional capacity in this population. Only weak correlations were identified between the traditional markers of left ventricular systolic function and exercise parameters. Although functional impairment and left ventricular systolic dysfunction are present in the advanced stages of cardiomyopathy, the reasons for the lack or weak association between LVEF and VO2peak is unclear. A reduction in functional capacity may precede the left ventricular systolic dysfunction^7^. Therefore, electrical conduction disorders in the early stages of heart disease lead to significant myocardial damage^34^ that reduces cardiac output, limiting the duration of ventricular filling, increasing myocardial oxygen demand, and potentially contributing to exercise intolerance. In contrast, left ventricular systolic dysfunction is detected over years due to the inflammatory process, myocarditis, reparative fibrosis, and extracellular matrix alterations^35^.

A previous study^36^ emphasized the role of left ventricular diastolic function such as E/e’ ratio as an important determinant of exercise intolerance in cardiac patients. The effect of left ventricular diastolic dysfunction on cardiac output may explain its association with functional capacity. During exertion, abnormalities in diastolic relaxation and left ventricular filling may result in filling rates below the required cardiac output even if the ventricular systolic properties are normal^37^. Inadequate cardiac output due to left ventricular filling can negatively affect exercise tolerance. Corroborating this hypothesis, Nunes et al.^27^ demonstrated that left ventricular diastolic function was not an independent determinant of functional capacity in patients with ChC without elevated left ventricular filling pressures (median E/e’ ratio=8).

One study^27^ including more variables assessed on echocardio-graphy and a larger sample than those in other studies found that age, sex, and right ventricle systolic velocity were associated with VO2peak. Together, these variables explained 71% of the variance in VO2peak. This study was the first to demonstrate the close relationship between the right ventricle and functional capacity being stronger than the systolic and diastolic functions of the left ventricle. The association between right ventricular systolic function and functional capacity were related to the elevation of pulmonary wedge pressure during exercise^27^, which leads to increased pulmonary resistance and consequently reduced right ventricular ejection and cardiac output during exercise^27^ ^,^ ^38^.

## THE PROGNOSTIC VALUE OF MAXIMAL EXERCISE TESTS

Eight of the included articles verified the prognostic significance of maximal exercise tests (Table 2). Due to the fatigue and dyspnea experienced by ChC patients, especially those with severely impaired systolic function and cardiac chamber dilation, the importance of functional assessment of these patients by exercise tests is undeniable. However, unlike heart failure due to other etiologies, little information is available regarding the prognostic value of the parameters evaluated during exertion in patients with ChC.

**TABLE 2:** Prognostic value of Maximal Exercise Tests in patients with ChC in the included studies (n=8).

STUDY	POPULATION	EXERCISE TEST	RESULTS
De Paola et al. (1995)^43^	69 patients with ChC (dilated and preserved LVEF), 46±12 years; 54% male; NYHA I-III, LVEF 46.6±18.6%. Follow-up period: 24±15 months. Endpoint: sudden death	Maximal Exercise Test (Bruce protocol, treadmill)	The number of patients with ventricular tachycardia at baseline was significantly higher in the sudden cardiac death group when compared to survivors (p<0.05).
Silva et al. (2017)^41^	45 patients with dilated ChC, 50.24±10.79 years; 100% male. Endpoint: death	CPET (cycle ergometer)	The VO2peak AT is an independent predictor of death (AUC=0.706). When both Rassi score and AT were defined as independent variables. VO2peak AT increases the accuracy of the Rassi score for mortality prediction by 5%.
Souza et al. (2015)^42^	21 patients with dilated ChC, 54.5±11.9 years, 38.1% male, NYHA III-IV, LVEF 29.2± 6.0%. Follow-up period: 24 months. Endpoint: cardiac death	CPET (ramp protocol, treadmill)	Differences between non-survivors ChC compared to survivors included lower peak HR (p=0.026), peak SBP (p=0.038), VO2peak (p=0.043), VO2peak AT (p=0.016), circulatory power (p=0.006) and ventilatory power (p=0.008). In the logistic regression, only circulatory power was independently associated with survival (OR 17.3 [95% CI: 1.39 to 217.0]). The circulatory power showed good accuracy in identify mortality (cut-off point ≤ 1280).
Ritt et al. (2013)^13^	55 patients with dilated ChC, 52±9 years, 69% male, NYHA II-IV, LVEF 27.6±6.6%. Follow-up period: 32±19 months. Endpoint: cardiac death	CPET (ramp protocol, treadmill)	The VO2peak (p=0.03) and VE/VCO2 slope (p=0.01) differed significantly between survivors and non-survivors. The VO2peak was correlated with MLwHRQ (r= -0.301; p=0.02) and showed good accuracy in identifying mortality (cut-off ≤18 mL/kg/min). The VE/VCO2 slope showed good accuracy in identifying mortality (cut-off >32.5. After adjusting for age, LVEF, and Chagas score, VE/VCO2 slope remained an independent predictor of mortality (adjusted HR: 2.80, 95% CI: 1.30 to 5.80, and p=0.001 for those with VE/VCO2 slope ≥32.5).
Pedrosa et al. (2011)^44^	130 patients with ChC (dilated and with preserved LVEF), 50.7±10.3 years, 40.8% male. Follow-up period: 9.9 years (range, 132 days to 17 years). Endpoint: cardiovascular death	CPET (Bruce protocol)	The prevalence of EIVA was 43.1%. Sex, age, and cardiothoracic index were not associated with EIVA. LVEF showed a statistically significant association with EIVA (p=0.01). The presence of EIVA alone was not a predictor of mortality but predicted mortality in Cox analysis, only when associated with age and cardiothoracic index >0.5 (hazard ratio=4.3 [95% CI: 1.6 to 11.4]; p=0.004).
Costa et al. (2018)^40^	49 patients with dilated ChC, 50±7 years; 57% male; NYHA I-III, LVEF: 36.0 [31.0-41.0]%. Follow-up period: 39±14 months. Endpoint: cardiac death	Maximal Exercise Testing (Bruce protocol, treadmill)	Survivors had higher VO2peak (p=0.048) than non-survivors. In the final model, VO2peak (hazard ratio 1.2, 95% CI: 1.0 to 1.3; p=0.009) remained an independent predictor of cardiac death in ChC. The optimal cut-off point for VO2peak in predicting death was 25 mL/kg/min. However, the established cutoff point failed to demonstrate a difference between the groups with VO2peak below and above 25 mL/kg/min.
Mady et al. (1994)^12^	104 patients with dilated ChC, 40.3±9.0 years; 100% male; NYHA II-IV, LVEF: 37.4±11.1%. Follow-up period: 41±12 months. Endpoint: cardiac death	CPET (Naughton protocol, treadmill)	The survivor group showed higher LVEF (=0.001), higher VO2peak (p=0.001), and better NYHA functional class (p=0.001) than that of those in the non-survivor group. In the multivariate model, VO2peak (p=0.001) and LVEF (p=0.008) remained independent predictors of cardiac death. Survival was significantly better in patients with VO2peak >20 mL/kg/min.
Costa et al. (2019)^39^	75 patients with ChC (dilated and with preserved LVEF), 48.4±8.0 years; 61% male; NYHA I-III, LVEF: 41.0 [35.0-53.5]%. Follow-up period: 41±12 months. Endpoint: death, heart transplantation, or ischemic event	Maximal Exercise Test (Bruce protocol, treadmill)	Patients with adverse events had lower LVEF (p=0.002), higher LVDD (p=0.019) and worse mental component of HRQoL (p=0.043) compared to those in patients without adverse events. No differences were observed in age, sex, NYHA functional class, VO2peak, %HR achieved during exercise test, and HR recovery after exercise testing between groups. In the univariate analysis, VO2peak, %HR achieved during exercise test, and HR recovery after exercise testing were not associated with adverse events.

Data presented as mean±standard deviation; mean [95% CI] or percentage. Abbreviations: **ChC:** Chagas cardiomyopathy; **NYHA:** New York Heart Association; **LVEF:** left ventricular ejection fraction; **CPET:** Cardiopulmonary Exercise Testing; **VO2peak:** peak oxygen uptake; **EIVA:** exercise-induced ventricular arrhythmias; **AT:** anaerobic threshold; **AUC:** area under the ROC curve; **ROC:** receiver operating curve; **MLwHFQ:** Minnesota Living with Heart Failure Questionnaire; **HRQoL:** health-related quality of life; **LVDD:** left ventricular end-diastolic diameter; **E/e´ ratio:** ratio of the early diastolic transmitral flow velocity to early diastolic mitral annular velocity; **VE/VCO2 slope:** minute ventilation/carbon dioxide production slope; 6MWT: six-minute walk test.

The prognostic values of VO2peak and VE/VCO2 slope in patients with heart failure are well-established^29^ ^-^ ^32^. Thus, they are also expected to have significance in predicting poor outcome in patients with ChC. Four studies evaluated the role of VO2peak in the prediction of adverse events. Costa et al.^39^ was the only study to include ChC patients with both dilated ChC and with preserved LVEF, showing that VO2peak was not associated with poor prognosis. However, the authors selected patients with a different clinical and functional profile and adopted different outcome criteria in addition to cardiac death. Patients demonstrate clinical heterogeneity even within the same disease stage. For prognostic purposes, stratifying patients into more homogeneous criteria could allow more reliable and robust analyses.

Ritt et al.^13^ verified that in patients with only dilated ChC, VO2peak showed good accuracy in identifying mortality. Patients with VO2peak ≤18 mL/kg/min had a mean survival of 29±3 months *versus* 46±5 months for those with VO2peak >18 mL/kg/min. However, after adjusting for age, LVEF, and Chagas score, VO2peak was no longer significantly associated with mortality. 

In patients with dilated ChC, Mady et al.^12^ demonstrated that only VO2peak and LVEF were independent predictors of death. The survival rate was significantly higher in patients with VO2peak >20 mL/kg/min. All patients with VO2peak values below 10 mL/kg/min died before 1 year. Similarly, another study^40^ that also evaluated the prognostic value of VO2peak in dilated ChC found that VO2peak was an independent predictor of death in patients with ChC (optimal cut-off: 25 mL/kg/min). However, the established cutoff point failed to differentiate between groups with VO2peak values below and above 25 mL/kg/min. We believe that VO2peak in dilated ChC is at least associated with a worse prognosis. However, due to the limited number of studies and the small sample sizes used, more studies are needed to confirm this hypothesis.

Only one study^13^ assessed the prognostic significance of the VE/VCO2 slope and reported good accuracy in identifying mortality risk. Patients with a VE/VCO2 slope ≥32.5 had a mean survival of 28±3 months *versus* 47±5 months for those with a slope of <32.5. After adjusting for age, LVEF, and Chagas score, VE/VCO2 slope remained an independent predictor of mortality. Therefore, this variable may be a valuable marker of worse outcomes in dilated ChC.

The prognostic values of two other variables assessed using CEPT were also verified. VO2peak AT was identified as an independent predictor of death in patients with dilated ChC^41^ and circulatory power showed good accuracy in identifying the risk of mortality^42^.

EIVA may also be a finding of prognostic importance in this population as ventricular arrhythmias during exertion may be detectable even in asymptomatic patients. One study^43^ reported a significantly higher number of patients with ventricular tachycardia during exercise test at baseline in the sudden cardiac death group than that in the survivor group. Another study^44^ evaluating the prognostic value of EIVA in patients with ChC (dilated and with preserved LVEF) demonstrated that the presence of EIVA alone was not a predictor of mortality. However, the presence of EIVA can predict mortality when associated with age and cardiothoracic index >0.5.

## THE ROLE OF FIELD TESTS IN FUNCTIONAL EVALUATION

Although the CPET is the gold standard for measuring exercise capacity, field tests have emerged as valuable tools for patients unable to perform maximal exercise tests or in places where the CPET is not available. Among field tests, the 6MWT and Incremental Shuttle Walk Test (ISWT) are most often used for patients with heart and pulmonary diseases^45^.

The 6MWT is an easy-to-perform test and is well-tolerated by patients. Patients are instructed to walk as far as possible in six minutes along a corridor (30 meters), without running^46^. The outcome is the final distance. However, in the 6MWT, patients tend to select a comfortable speed and not stress themselves with a maximal effort^47^.

Compared to the 6MWT, the ISWT employs a standardized methodology and a progressive character that is more similar to the maximum tests. Patients are required to walk at a defined speed in a 10-meter corridor, as dictated by a series of beeps from an audio recorder^48^. The walking speed is progressively increased at 1-min intervals for a total of 12 stages. The test ends when the patient fails to complete a shuttle in the time required. The target outcome is the final distance.

Field tests are widely used in patients with ChC and can provide valuable information about the functional status of the patient. The present review included 10 articles that applied field tests in ChC. In the 6MWT, the distance walked was positively correlated with VO2peak^23^ ^,^ ^26^ ^,^ ^49^, LVEF^50^, hemoglobin levels^51^, inspiratory muscle strength^52^, and some Short-form Health Survey (SF-36) domains^53^. Furthermore, the 6MWT was correlated with VO2peak in both dilated ChC and with preserved LVEF^23^, although the correlation was stronger in the dilated group. 

Moreover, the 6MWT distance was inversely correlated with levels of blood biomarkers, including brain natriuretic peptide (BNP) levels^50^ ^,^ ^53^ and monocyte chemoattractant protein-1 (MCP-1)^50^ as well as some echocardiographic features^53^. The distance was strongly correlated with health-related quality of life evaluated by the Minnesota Living with Heart Failure Questionnaire (MLwHRQ)^13^ ^,^ ^51^ ^,^ ^53^. The 6MWT distance was the only independent determinant of MLwHRQ score. The presence of systemic arterial hypertension did not reduce the functional capacity of patients with ChC^51^.

Costa et al^26^ developed and validated an equation based on sex, NYHA class, 6MWT distance, age, and body mass index to predict the VO2peak evaluated by gas analysis in patients with ChC (R^2^=0.61).

Several studies evaluated the prognostic value of the 6MWT.^13^ ^,^ ^54^ Ritt et al.^13^ reported differences in the distances between survivors and non-survivors. However, the test did not provide accurate information on the mortality of patients with dilated ChC. Similarly, Costa et al.^54^ also found no prognostic value of the 6MWT distance in 60 patients with dilated ChC.

The ISWT is another test widely used for functional assessment of patients with heart diseases. In ChC and samples comprising dilated and preserved LVEF, the ISWT distance was positively correlated with VO2peak^49^ ^,^ ^55^ ^,^ ^56^ and some SF-36 domains^56^. Significant negative correlations were reported between ISWT distance and VE/VCO2 slope^49^ and MLwHRQ score^56^.

Prediction equations have been proposed to estimate the VO2peak assessed using CPET with ISWT^55^. The equations are based on the ISWT distance, NYHA class, and sex (Table 3). Unfortunately, no study has verified the prognostic significance of the test in predicting adverse events in patients with ChC.

**TABLE 3:** Functional and prognostic evaluation in patients with ChC by field tests (n=10)

STUDY	POPULATION	EXERCISE TESTS	RESULTS
Sousa et al. (2008)^50^	38 patients with dilated ChC, 48±10 years; 68% male; NYHA I-III, LVEF<55%	6MWT	The 6MWT distance was correlated with MCP-1 values (r=−0.358, p=0.04), BNP levels (r=−0.349, p=0.04), and LVEF (r=0.451, p=0.004) but not with NYHA functional class (r=−0.130, p=0.435).
Dourado et al. (2010)^51^	60 patients with ChC: 55±14 years; 68% male; 25% in NYHA III-IV; LVEF: 44.0±13.8% and 38 patients with ChC and systemic arterial hypertension: 63±10 years; 88% male; 21% in NYHA III-IV; LVEF: 51.8±12.9%	6MWT	No difference in 6MWT distance between groups with and without systemic arterial hypertension (p>0.05). In the systemic arterial hypertension group, the 6MWT distance was correlated with MLwHFQ (r=-0.51; p=0.001). In the group without systemic arterial hypertension, the 6MWT distance was correlated with hemoglobin levels (r=0.34; p=0.007) and MLwHFQ (r= -0.38; p=0.003).
Ritt et al. (2013)^13^	55 patients with dilated ChC (52±9 years, 69% male, NYHA II-IV, LVEF: 27.6±6.6%). Follow-up period: 32±19 months; endpoint: cardiac death	CPET (ramp protocol, treadmill) and 6MWT	The 6MWT was correlated with MLwHRQ (r=-0.375; p=0.007) and was the only independent determinant of MLwHRQ (each 10-min increase in distance walked was associated with a 0.7-point reduction in MLHFQ score); no prognostic value.
Costa et al. (2014)^56^	35 patients (dilated and preserved LVEF), 47.1±8.2 years, 66% male, NYHA I-III and LVEF: 59 (41-64)%	CPET (ramp protocol, treadmill) and ISWT	ISWT distance was correlated with VO2peak (r=0.587; p<0.001), MLwHRQ score (r=-0.460; p=0.006), and SF-36 domains physical functioning (r=0.435; p=0.009), role physical (r=0.447; p=0.008), and mental health (r=0.430; p=0.011).
Costa et al. (2014)^23^	41 patients with ChC (dilated and with preserved LVEF), 47.8±8.3 years; 68% male; NYHA I-III	CPET (ramp protocol, treadmill) and 6MWT	Patients with dilated ChC showed lower 6MWT distance (p=0.045) compared to that in patients with preserved LVEF. The 6MWT distance was correlated with VO2peak (r=0.586; p<0.001) but not with VE/VCO2 slope (r=−0.046; p=0.776). The 6MWT distance was correlated with VO2peak in both dilated ChC (n=20, r=0.612; p=0.005) and preserved LVEF (n= 21, r=0.463; p=0.035) groups.
Alves et al. (2016)^55^	32 patients with ChC (6 with dilated ChC and 26 with preserved LVEF), 58.8±9.0 years; 18.7% male; NYHA I-III, LVEF: 62.4±13.4%	CPET (Bruce protocol, treadmill) and ISWT	The ISWT distance was correlated with VO2peak (r=0.456; p=0.009). In women, the VO2peak was predicted by the formula 13.97 + 0.02 x ISWT distance (for NYHA I) or 11.36 + 0.02 x ISWT distance (for NYHA³II). In men, the VO2peak was predicted by the formula 12.21 + 0.03 x ISWT distance (for NYHA I) or 9.60 + 0.03 x ISWT distance (for NYHA³II).
Chambela et al. (2017)^53^	40 patients with dilated ChC, 60±12 years; 47% male; NYHA I-III, LVEF: 35±12%	6MWT	The 6MWT distance was correlated with BNP (r=-0.37; p=0.02) and echocardiographic features, including E velocity (r=-0.38; p=0.002), E/E’ ratio (r=-0.32; p=0.05), LV diastolic dysfunction (r=-0.36; p=0.03), mitral regurgitation (r= -0.53; p<0.001), and PASP (r=-0.42; p=0.02). The 6MWT distance was also correlated with the SF-36 domains physical functioning (r=0.46; p=0.008), physical role functioning (r=0.37; p=0.04), and bodily pain (r=0.43; p=0.014) as well as MLwHRQ (r=-0.54; p=0.002).
Costa et al. (2017)^26^	81 patients with ChC (dilated and with preserved LVEF), 48.6±8.1 years; 63% male; NYHA I-III, LVEF: 43.7±13.7%	Maximal Exercise Test (treadmill) and 6MWT	The VO2peak was correlated with the 6MWT distance (r=0.527; p<0.001) and VO2peak was predicted by the formula 53.43 + (1.35 × sex) - (5.59 × NYHA) + (0.01 × 6MWT distance) - (0.29 × age) - (0.035 × BMI).
Costa et al. (2017)^54^	60 patients with dilated ChC, 52.6±9.4 years; LVEF: 27.1±5.5%. Follow-up period: 7.5 years. Endpoint: death	6MWT	The 6MWT was not a predictor of death. The independent predictors of death were non-sustained ventricular arrhythmias in 24h Holter monitoring and left atrium volume index (p<0.05 for both).
Costa et al. (2018)^49^	35 patients with ChC (dilated and with preserved LVEF), 47.1±8.2 years; 66% male; NYHA I-III, LVEF: 59.0 [41.0-64.0]	CPET (ramp protocol, treadmill), 6MWT and ISWT	The VO2peak was correlated with 6MWT distance (r=0.577; p<0.001) and ISWT distance (r=0.587; p<0.001). Only the ISWT was correlated with the VE/VCO2 slope (r=-0.339; p=0.003). The optimal distances to identify patients with functional impairment were 520 m for the 6MWT and 400 m for the ISWT.

Data presented as mean±standard deviation; median (25-75%); mean [95% CI] or percentage. Abbreviations: **ChC:** Chagas cardiomyopathy; **NYHA:** New York Heart Association; **LVEF:** left ventricular ejection fraction; **6MWT:** six-minute walk Test; **MLwHFQ:** Minnesota Living with Heart Failure Questionnaire; **CPET:** Cardiopulmonary Exercise Testing; **VO2peak:** peak oxygen uptake; **VE/VCO2 slope:** minute ventilation/carbon dioxide production slope; **HRQoL:** health-related quality of life; **SF-36:** Short-form of Health Survey; **ISWT:** Incremental Shuttle Walk Test; **BNP:** brain natriuretic peptide; **E velocity:** peak early diastolic filling velocity; **E/E´ ratio:** ratio of the early diastolic transmitral flow velocity to early diastolic mitral annular velocity; **PASP:** pulmonary artery systolic pressure; **AUC:** area under the ROC curve; **MCP-1:** monocyte chemoattractant protein-1.

Finally, one study^49^ compared the distances walked between field tests (6MWT and ISWT) and the accuracy of both in the identification of patients with functional impairment (VO2peak <20 mL/kg/min). No significant differences were observed between the distances walked. Furthermore, the optimal distances to identify patients with functional impairment were 520 m for the 6MWT and 400 m for the ISWT.

## CURRENT CHALLENGES

Chagas disease persists as an important and neglected cause of loss of years of healthy life due to premature mortality and disability^57^; furthermore, robust clinical trials are scarce in Chagas disease and the recommendations are often based on guidelines for other cardiopathies^58^ ^,^ ^59^.

Moreover, few studies have verified the effectiveness of the variables assessed using the exercise tests in predicting patient survival. The VO2peak is an important criterion for heart transplantation in patients with ChC^60^; however, its prognostic role should be better understood in the context of preventive strategies, risk stratification, and early diagnosis. In addition, it is necessary to establish strong cut-off points or deepen the recognition of existing ones. The criteria proposed by Weber et al.^16^ are well-founded but directed at patients with non-ChC heart failure and it remains unknown if they are effective in ChC. In patients with ChC and preserved LVEF, is the exercise test useful only for the assessment of EIVA, chronotropic incompetence, and ischemia, or can some functional variables contribute to signal an unfavorable prognosis in the medium or long term?

Chagas is a neglected tropical disease for which many studies are performed in endemic areas, usually with low human development indexes, limited resources, and few technological devices. Thus, studies on functional assessment using field tests should be encouraged. However, some questions remain unanswered. For instance, can field tests provide prognostic information? Are both 6MWT and ISWT safe for patients or can they trigger or exacerbate ventricular arrhythmias? Which of the field tests is more effective in identifying patients with poor outcomes? Do they predict survival in a wide spectrum of patients with ChC or just a subgroup according to systolic function? What is the responsiveness of the field tests?

Finally, with the population aging, elderly patients with infection deserve special attention because they are a vulnerable group owing to the combination of Chagas disease and chronic-degenerative comorbidities^61^. Several studies have targeted this population. Elderly patients with Chagas disease usually present the cardiac form^61^ ^-^ ^63^, and comorbidities are detected in approximately 60% of patients^62^. Furthermore, elderly patients with ChC have lower VO2peak values and a higher prevalence of ventricular arrhythmias than do younger patients with the same clinical presentation^64^. Therefore, studies addressing functional evaluation and exercise prescription in situations with the coexistence of ChC and comorbidities, including hypertension, osteoporosis, osteoarthritis, dyslipidemia, and diabetes, are required.

## FINAL CONSIDERATIONS

In patients with ChC, the VO2peak was correlated with many demographic, clinical, and echocardiographic variables. The main echocardiographic determinants included left ventricular diastolic function and right ventricular systolic velocity. In addition, VO2peak was associated with higher mortality. The VE/VCO2 slope emerged as a potential prognostic measure evaluated by the CPET. Finally, both field tests (6MWT and ISWT) demonstrated efficacy in the functional evaluation of patients with ChC. However, the prognostic value of the tests remains unknown and further studies are needed, considering their low operational costs and the setting of Chagas disease.
